# Clinical and genetic characteristics of Chinese patients diagnosed with chronic enteropathy associated with *SLCO2A1* gene

**DOI:** 10.1186/s13023-024-03177-y

**Published:** 2024-05-16

**Authors:** Qing Shang, Yimin Dai, Jingyi Huang, Wei Liu, Weixun Zhou, Yaping Liu, Hong Yang, Qiang Wang, Yue Li

**Affiliations:** 1 Department of Gastroenterology, Peking Union Medical College Hospital, Chinese Academy of Medical Sciences and Peking Union Medical College, Beijing, 100730 China; 2 School of Medicine, Tsinghua University, Beijing, 100084 China; 3 Department of Radiology, Peking Union Medical College Hospital, Chinese Academy of Medical Sciences and Peking Union Medical College, Beijing, 100730 China; 4 Department of Pathology, Peking Union Medical College Hospital, Chinese Academy of Medical Sciences and Peking Union Medical College, Beijing, 100730 China; 5 McKusick-Zhang Center for Genetic Medicine, State Key Laboratory of Medical Molecular Biology, Institute of Basic Medical Sciences Chinese Academy of Medical Sciences, School of Basic Medicine Peking, Union Medical College, Beijing, 100730 China

**Keywords:** SLCO2A1, Chronic enteropathy associated with *SLCO2A1* gene, Chinese

## Abstract

**Background and aims:**

Chronic enteropathy associated with *SLCO2A1* gene is a rare intestinal disease caused by loss-of-function *SLCO2A1* mutations, with clinical and genetic characteristics remaining largely unknown, especially in Chinese patients. This study aims to reveal clinical and genetic features of Chinese CEAS patients, highlighting the previously unreported or unemphasized characteristics.

**Methods:**

We enrolled 12 Chinese patients with chronic enteropathy associated with *SLCO2A1* gene admitted to Peking Union Medical College Hospital from January 2018 to December 2022. Clinical and genetic data of these patients were collected and analyzed.

**Results:**

58.3% of patients were male, who also had primary hypertrophic osteoarthropathy, whereas female patients did not have primary hypertrophic osteoarthropathy. Apart from common symptoms associated with anemia and hypoalbuminemia, abdominal pain, ileus, diarrhea, and hematochezia were present. 4 of the 5 female patients had early-onset amenorrhea, though the causal relationship remained to be clarified. Endoscopy and computed tomography enterography revealed that lesions can occur in any part of the digestive tract, most commonly in the ileum. Pathology showed multiple superficial ulcers with adjacent vascular dilatation, and loss of SLCO2A1 expression, particularly in gastrointestinal vascular endothelial cells. Genetic analysis confirmed *SLCO2A1* mutations in all patients and identified 11 new *SLCO2A1* variants for CEAS.

**Conclusions:**

This study reports new clinical, pathological, and genetic findings in 12 Chinese patients with chronic enteropathy associated with *SLCO2A1* gene. This study provides insights into the pathogenesis of this disease. However, studies with larger sample sizes and more in-depth mechanism research are still required.

**Supplementary Information:**

The online version contains supplementary material available at 10.1186/s13023-024-03177-y.

## Introduction

Chronic enteropathy associated with *SLCO2A1* gene (CEAS) is a rare monogenic disease characterized by multiple intestinal ulcers due to loss-of-function mutations in *SLCO2A1* gene. CEAS was initially recognized as chronic nonspecific multiple ulcers of the small intestine (CNSU) based on histology [1]. After identifying *SLCO2A1* mutations as the genetic etiology of CNSU, the term “chronic enteropathy associated with *SLCO2A1* gene” was proposed [2].

SLCO2A1, short for solute carrier organic anion transporter family member 2A1, functions to mediate the transmembrane transport of prostaglandins (PGs). In addition to CEAS, SLCO2A1 defects can cause a subtype of primary hypertrophic osteoarthropathy (PHO), PHO autosomal recessive 2 (PHOAR2) featuring digital clubbing, pachydermia, and periostosis [3].

Studies of Japanese and Korean CEAS patients show CEAS predominantly affects females, and a portion of male CEAS patients suffer from PHO-related symptoms concurrently [4, 5]. Intestinal ulcers caused persistent loss of blood and protein, so most CEAS patients displayed iron deficiency anemia and hypoalbuminemia [4–6]. Endoscopy often detects multiple small intestinal lesions, more commonly in the ileum [4–6]. Genetic analysis has identified an increasing number of novel *SLCO2A1* mutations in CEAS patients [2, 7, 8]. In vitro functional analysis confirmed the impaired prostaglandin E2 (PGE2) transport ability of 10 SLCO2A1 variants found in CEAS patients [9]. Recently, Chinese CEAS cases with PHO were reported, showing multiple intestinal ulcers and segmental stenosis [10, 11]. In addition to patients of Asian origins, the first French Caucasian female siblings with CEAS sharing the same compound heterozygous *SLCO2A1* mutations were reported in 2022, suggesting CEAS may be more prevalent and widespread than previously thought [12].

Nevertheless, due to the relatively late recognition and definition of CEAS, as well as the small number of confirmed cases, our understanding of the clinical characteristics, especially pathological and genetic characteristics of this disease remains largely insufficient. This study aims to describe the clinical and genetic features of 12 Chinese CEAS patients admitted to Peking Union Medical College Hospital from January 2018 to December 2022, emphasizing unique pathological and genetic characteristics not previously reported. We aim to improve understanding of CEAS characteristics and provide a basis for further mechanism studies.

## Materials and methods

### Subjects and clinical data

We enrolled 12 patients diagnosed with CEAS at Peking Union Medical College Hospital from January 2018 to December 2022. The CEAS diagnosis was based on acknowledged clinical criteria and genetic identification of *SLCO2A1* gene variants. We collected demographic and clinical data including gender, age at CEAS diagnosis, age at symptom onset, past medication use, treatment history and effect, family history of CEAS and PHO, parental consanguinity, CEAS and PHO symptoms, endoscopic findings, surgical pathology, abdominal surgeries, and laboratory test results. PHO diagnosis was based on typical clinical manifestations including digital clubbing, pachydermia, and periostosis by X-ray (data not shown). The results of endoscopy, computed tomography enterography (CTE) and surgical pathology were interpreted by experienced specialists. Sites of gastrointestinal (GI) tract lesions were determined by endoscopy, CTE and surgical findings.

Informed consents were obtained from all subjects. This study was approved by the institutional review boards (IRB numbers: *S_K1478*).

### Genomic DNA preparation, whole exome sequencing and Sanger sequencing

Genomic DNA was extracted from patients’ peripheral blood. Whole exome sequencing and sanger sequencing was performed on Illumina sequencing platform, and the sequencing fragments were compared to reference genome hg19 to identify and further confirm the sequence and mutations of *SLCO2A1* of each subject.

### Immunohistochemical staining

Paraffin-embedded tissue samples were obtained from surgically excised tissue. Control samples were obtained from subjects without CEAS. Immunohistochemical (IHC) staining was performed on sections from paraffin-embedded samples using anti-SLCO2A1 polyclonal antibody (HPA013742, Atlas Antibodies) and anti-CD31 antibody (PA0414, LEICA). The sections were observed and the extent of SLCO2A1 expression was evaluated under microscope. Pictures were taken with CaseViewer Software (version 2.4, 3DHISTECH Ltd).

### Statistical analysis

Summary categorical variables were expressed as numbers or numbers with percentages. Individual categorical variables were labeled as “-” (negative), “+” (positive) or “/” (not available). Summary continuous variables were shown as medians with interquartile ranges (IQRs). Genetic data was analyzed with Polyphen2, ClinVar and MutationTaster to estimate pathogenicity of the variants. Human gene mutation database (HGMD) was used to search for mutation-related information.

## Results

### Clinical features

Of the 12 CEAS patients enrolled from January 2018 to December 2022, 7 (58.3%) were male (Tables 1 and 2). The median age at CEAS symptom onset was 19 (IQR, 10-24) years old, while the median age at CEAS diagnosis was 32 (IQR, 29-40) years old. The median interval from symptom onset to diagnosis was 11.5 (IQR, 9.5-24.5) years. Seven males (58.3%) all met the diagnosis of PHO, but none of the females did. Five patients (41.7%) reported a short-term use of nonsteroidal anti-inflammatory drugs (NSAIDs). No patient had a family history of CEAS. However, 3 patients (25.0%) had family history of PHO in their male relatives. Four patients (33.3%) had parental consanguinity.

**Table 1:** Clinical characteristics of 12 patients diagnosed with CEAS

**Characteristics**	***n*=12**
Gender, Male, n(%)	7(58.3)
Age at CEAS diagnosis, median (IQR), yr	32(29, 40)
Age at CEAS symptom onset, median (IQR), yr	19(10, 24)
Interval from symptom onset to diagnosis, median (IQR), yr	11.5(9.5, 24.5)
PHO diagnosis, n(%)	7(58.3)
Gender, Male, n(%)	7(100.0)
Past NSAIDs use, n(%)	5(41.7)
Family history of CEAS, n(%)	0(0)
Family history of PHO, n(%)	3(25.0)
Parental consanguinity, n(%)	4(33.3)
**CEAS symptoms, n(%)**	
Abdominal pain	12(100.0)
Ileus	8(66.7)
Lower Limb Edema	7(58.3)
Diarrhea	5(41.7)
Melena	4(33.3)
Hematochezia	4(33.3)
Hypoalbuminemia	9(75.0)
Fever	2(16.7)
Pyloric obstruction	2(16.7)
**PHO symptoms, n(%)**	*n*=7
Digital clubbing	7 (100.0)
Pachydermia	7 (100.0)
Periostosis	7 (100.0)
BMI, median (IQR), kg/m^2^	17.9(16.8, 19.3)
**GI tract Distribution, n(%)**	
Multiple Lesions	12(100.0)
Esophagus	2(16.7)
Stomach	6(50.0)
Small Intestine	11(91.7)
Duodenum	4(33.3)
Jejunum	2(16.7)
Ileum besides terminal ileum	11(91.7)
Terminal ileum	6(50.0)
Colon	3(25.0)
Rectum	1(8.3)
**Endoscopic manifestations, n(%)**	
Reflux esophagitis	2(16.7)
Gastric polyps	5(41.7)
Hypertrophic gastritis	2(16.7)
Gastric anastomotic ulcer	2(16.7)
Multiple ileal strictures with annular ulcer	11(91.7)
Sigmoid colon stricture with annular ulcer	2 (16.7)
Diffuse mucosal erosions in colon and rectum	1(8.3)
**Surgical pathology manifestation, n(%)**	***n*=6**
Multiple superficial ulcers	6(100.0)
Blood vessel dilation and congestion	4(66.7)
Submucosal fibrotic proliferation	5(83.3)
**History of abdominal surgery, n(%)**	**9(75.0)**
Once	6(50.0)
Twice	3(25.0)
**Types of abdominal surgery, n(%)**	
Partial ileal resection	8(66.7)
Subtotal Gastrectomy	2(16.7)
Duration from CEAS symptom onset to surgery, year, median(IQR)	10(6, 14)
**Laboratory tests, median (IQR)**	
Lowest hemoglobin, g/L	67(49, 81)
Lowest albumin, g/L	28(22, 33)
CRP, mg/L	12.2(6.7, 34.9)
Platelet, 10^9^/L	259(185, 421)
Positive ASCA, n(%)	2(16.7)
Positive ANCA, n(%)	1(8.3)
**Treatment history, n(%)**	
Prednisone	7(58.3)
Etoricoxib	4(33.3)
Enteral nutrition	9(75.0)
Iron supplementation	12(100.0)
Mesalazine	7(58.3)
Endoscopic balloon dilation	2(16.7)
Thalidomide	2(8.3)

*CEAS* Chronic enteropathy associated with SLCO2A1 gene, *IQR* Interquartile range, *yr* year, *PHO* primary hypertrophic osteoarthropathy, *NSAIDs* Nonsteroidal anti-inflammatory drugs, *BMI* Body mass index, *GI* Gastrointestinal, *CRP* C-reactive protein, *ASCA* Anti-*Saccharomyces cerevisiae* antibodies, *ANCA* anti-neutrophil cytoplasmic antibodies

**Table 2:** Genetic Analysis of 12 CEAS patients

**Indexes**	**Chromosomal** **Location**	**Gene Location**	**Mutation Type**	**Change of Nucleotide**	**Mutation Site Type**	**Change of Amino Acids**	**Polyphen2 Prediction**	**ClinVar Prediction**	**MutationTaster** **Prediction**	**HGMD Reported**	**Other CEAS** **Reported**
Patient 1	133654625	E13	Homo	c.1807C>T	NON	p.R603X	NA	Pathogenic	Disease Causing	Yes	Yes
Patient 2	133670058	E6	Homo	c.855delA	FS	p.A286QfsX35	NA	Likely pathogenic	Disease Causing	Yes	No
Patient 3	133667748	E7	Homo	c.929G>A	MIS	p.D310G	Benign	Not Reported	Disease Causing	No	No
Patient 4	133666289	E9	Compound Hetero	c.1106G>A	MIS	p.G369D	Probably Damaging	Likely pathogenic	Disease Causing	Yes	No
	133667545	I7/E8		c.941-1G>A	SS	NA	NA	Likely pathogenic	NA	Yes	Yes
Patient 5	133666218	E9	Compound Hetero	c.1177delT	FS	p. S393Lfs*8	NA	Likely pathogenic	Disease Causing	Yes	No
	133664025	E10		c.1375T>C	MIS	p. C459R	Probably Damaging	Not Reported	Disease Causing	Yes	No
Patient 6	133657303	E12	Hetero	c.1660G>A	MIS	p.G554R	Probably Damaging	Pathogenic	Disease Causing	Yes	Yes
Patient 7	133667545	I7/E8	Compound Hetero	c.941-1G>A	SS	NA	NA	Likely pathogenic	NA	Yes	Yes
	133673814	E4		c.621C>A	NON	p.Y207X	NA	Pathogenic	Disease Causing	No	No
Patient 8	133666259	E9	Compound Hetero	c.1136G>A	MIS	p.G379E	Probably Damaging	Not Reported	Disease Causing	Yes	No
	133657282	E12		c.1681C>T	MIS	p. R561C	Probably Damaging	Not Reported	Disease Causing	Yes	Yes
Patient 9	133654661	E13	Homo	c.1771C>T	NON	p.R591X	NA	Not Reported	Disease Causing	Yes	No
Patient 10	133666288	I8/E9	Homo	c.1106-1G>A	SS	NA	NA	Not Reported	NA	Yes	No
Patient 11	133666289	E9	Homo	c.1106G>A	MIS	p.G369D	Probably Damaging	Likely pathogenic	Disease Causing	Yes	No
Patient 12	133698348	E2	Homo	c.211G>C	MIS	p.G71R	Probably Damaging	Not Reported	Disease Causing	Yes	No

*E* Exon, *I* Intron, *Homo* Homozygousm, *Hetero*, Heterozygous, *MIS* Missense, *NON* Nonsense, *SS* Splicing site, *FS* Frame shift, *NA* Not available, *HGMD* Human gene mutation database

All 12 patients presented with abdominal pain. Eight (66.7%) patients had ileus, and 2 (16.7%) had pyloric obstruction. Five (41.7%) patients suffered from diarrhea, 4 (33.3%) had melena, and 4 (33.3%) had hematochezia. Two (16.7%) patients had fever. The 7 male patients with PHO showed digital clubbing, pachydermia, and periostosis. Four of the 5 female patients developed early-onset amenorrhea. Regarding past medical history, one patient each suffered from congenital lymphatic dysplasia, cholelithiasis, and hypothyroidism respectively (Supplementary Table 1). The median body mass index (BMI) of the 12 patients was 17.9 (IQR, 16.8-19.3).

All 12 (100.0%) patients had multiple lesions, most commonly in ileum besides terminal ileum (91.7%). Other parts of the GI tract were also involved, from esophagus (16.7%), stomach (50.0%) to colon (25.0%) and rectum (8.3%), suggesting that SLCO2A1 plays an important role in the digestive tract and the pathogenicity of SLCO2A1 dysfunction is widespread along GI tract.

Laboratory tests revealed that the median lowest hemoglobin was 67 (IQR, 49-81) g/L and the median lowest albumin was 28 (IQR, 22-33) g/L, suggesting anemia and hyperalbuminemia. The median CRP was 12.2 mg/L (IQR, 6.7-34.9) mg/L. Two (16.7%) patients had positive anti-*Saccharomyces cerevisiae* antibodies (ASCA), and 1 (8.3%) patient had positive anti-neutrophil cytoplasmic antibodies (ANCA).

### Endoscopic and imaging characteristics

**Fig. 1:**
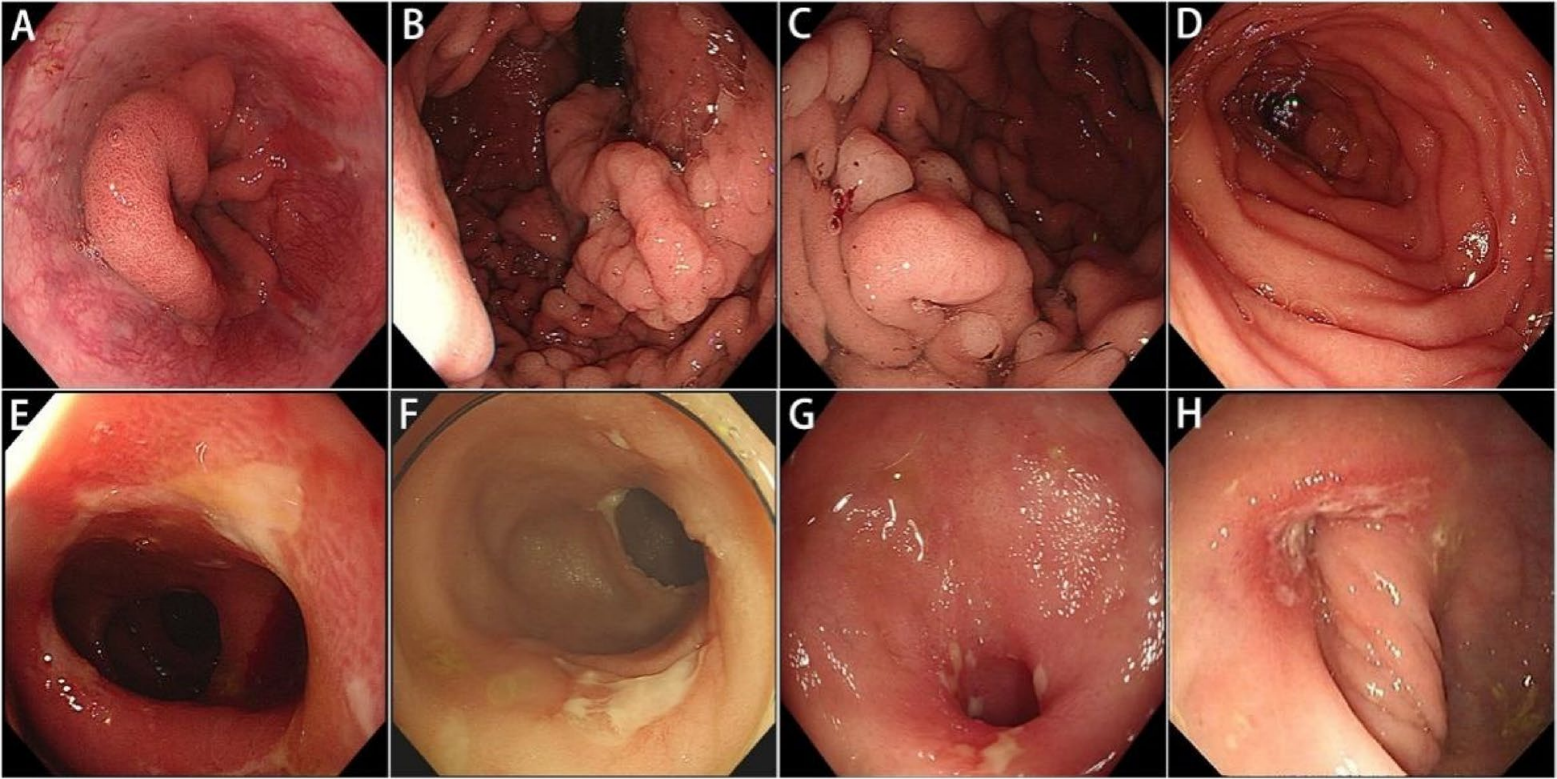
Endoscopic images of patients diagnosed with CEAS. **A-D**, gastroscopic pictures of patient 3, showing fold thickening in gastric cardia (**A**), fundus of stomach (**B**), gastric body (**C**), and descending duodenum (**D**). **E**, an enteroscopic picture of patient 7 showing stenosis and ulceration of the middle ileum. **F** and **G**, colonoscopic pictures of patient 6 (**F**) and patient 11 (**G**) respectively, showing circular stenosis and ulceration of the terminal ileum. **H**, a colonoscopic picture of patient 1 showing stenosis and ulceration of the sigmoid colon

Gastroscope, enteroscope and colonoscope were performed to locate and visualize the lesions along the GI tract in these patients. Statistically, multiple ileal strictures with annular ulcers were most common, present in 11 patients (91.7%). Besides, reflux esophagitis was present in 2 patients (16.7%), gastric polyps in 5 patients (41.7%), hypertrophic gastritis in 2 patients (16.7%), and gastric anastomotic ulcers in 2 patients (16.7%). Two patients (16.7%) showed sigmoid colon stricture with annular ulcer and 1 patient (8.3%) showed diffuse mucosal erosions in colon and rectum similar to clinical manifestations of ulcerative colitis (UC) (Table 1 and Supplementary Table 1). Representative endoscopic images were shown (Fig. 1). Gastroscopy of patient 3 showed smooth esophageal mucosa without erosive ulcer or varicose veins. However, the mucosal folds of the cardia, fundus, body of the stomach, and descending duodenum were thick (Fig. 1A-D). Furthermore, multiple polypoid eminences were present in his gastric body. The enteroscopy of patient 7 revealed scattered superficial ulcers, pseudodiverticula and multiple stenosis of group 5 small intestine (Fig. 1E). Colonoscopy of patient 6 and patient 11 revealed irregular superficial ulceration and circular superficial ulceration at the terminal ileum (Fig. 1F, G). Colonoscopy of patient 1 showed circular ulceration and symptomatic stricture of the sigmoid colon which called for balloon dilatation to release bowel obstruction (Fig. 1H).

**Fig. 2:**
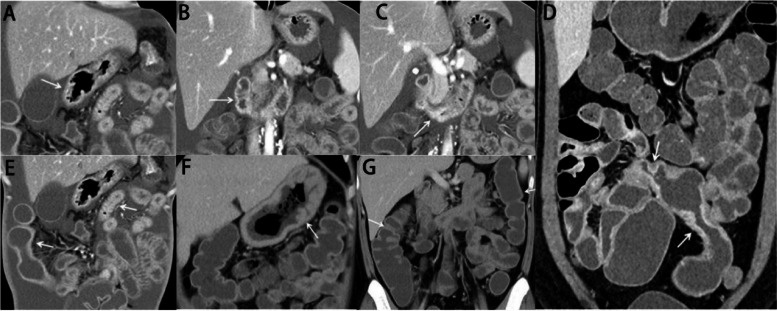
Representative CTE images of CEAS patients. **A-C**, computed tomography enteroscopy (CTE) images of patient 1, showing wall thickening of gastric antrum (arrow in **A**), descending duodenum (arrow in **B**) and horizontal duodenum (arrow in **C**) with enhanced mucosa. **D**, the CTE image of patient 11, showing thickened intestinal wall of multi-segmental ileum, enhanced mucosa, short-segmental stenosis (indicated by white arrows), and dilated intestinal lumen between strictured segments. **E**, the CTE image of patient 1 showing diffuse wall thickening of ileum with enhanced mucosa and multiple stenosis of ileum highlighted by white arrows. **F **and** G**, CTE images of patient 3, showing diffusely thickened gastric wall, thickened plicae and mildly enhanced gastric wall (arrow in **F**), and slightly thickened colon wall as well (arrows in **G**)

CTE was performed to help localize GI lesions mainly characterized by wall thickening, stenosis and mucosal enhancement. For example, CTE of patient 1 showed wall thickening of gastric antrum, descending duodenum, and horizontal duodenum with mucosal enhancement (Fig. 2A-C). Multiple ileum wall thickening with mucosal enhancement and stenosis were present in patient 11 and patient 1 as well (Fig. 2D and E). Intestinal segments were dilated between stenosis (Fig. 2D). The lesion sites could also be multiple in stomach and colon. In patient 3, CTE showed diffusely thickened gastric wall with mild enhancement, thickened plicae and slightly thickened colon wall (Fig. 2F and G).

### Surgical pathology characteristics

**Fig. 3:**
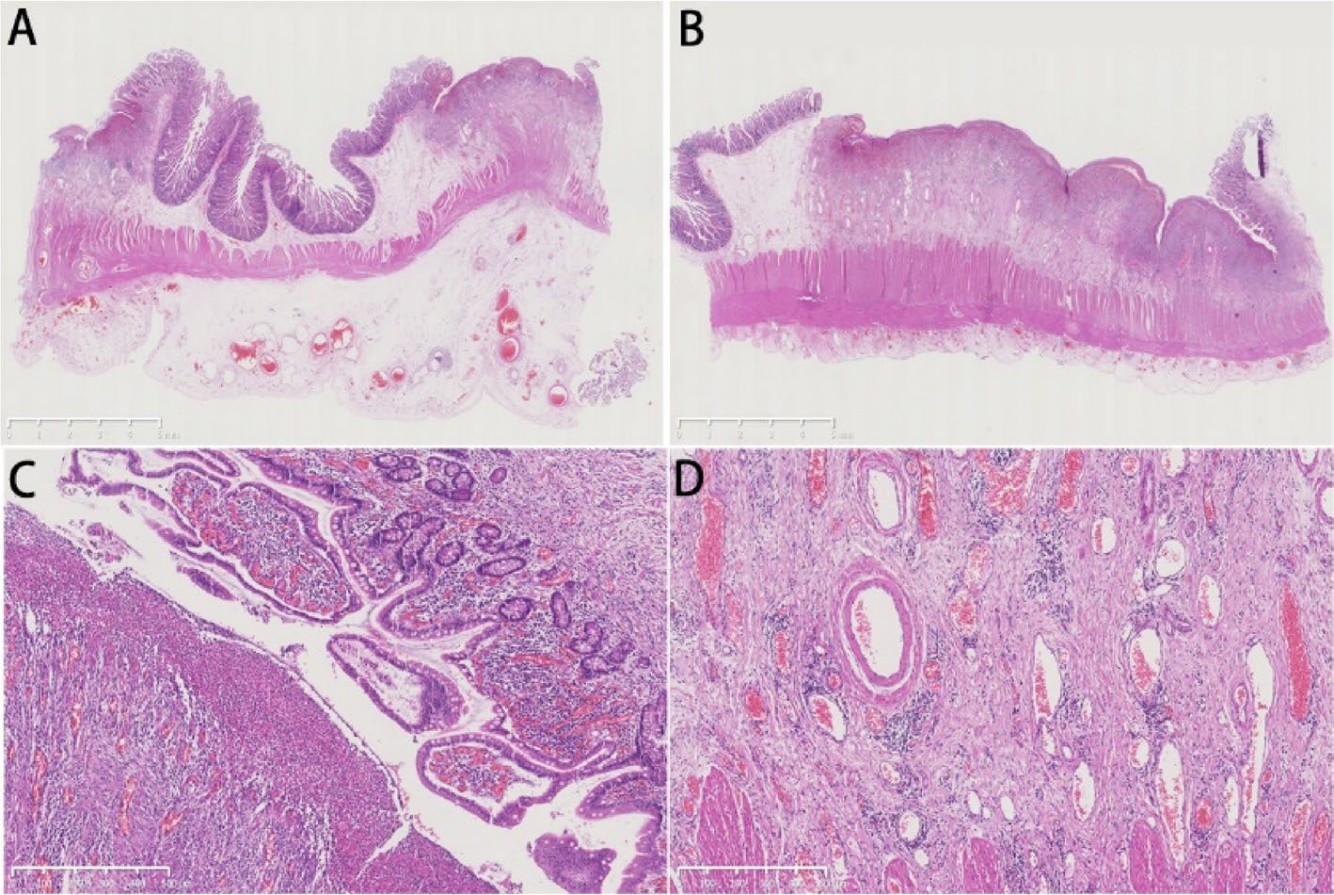
Histology of CEAS intraoperative pathological sections. Hematoxylin and Eosin staining. **A **and** B**, multiple superficial ulcers (scale bar, 5 mm). **C**, vasodilation and hyperemia of the small intestinal mucosa adjacent to ulcers (scale bar, 500 μm). **D**, hyperplasia of submucosal fibrous tissue (scale bar, 500 μm)

**Fig. 4:**
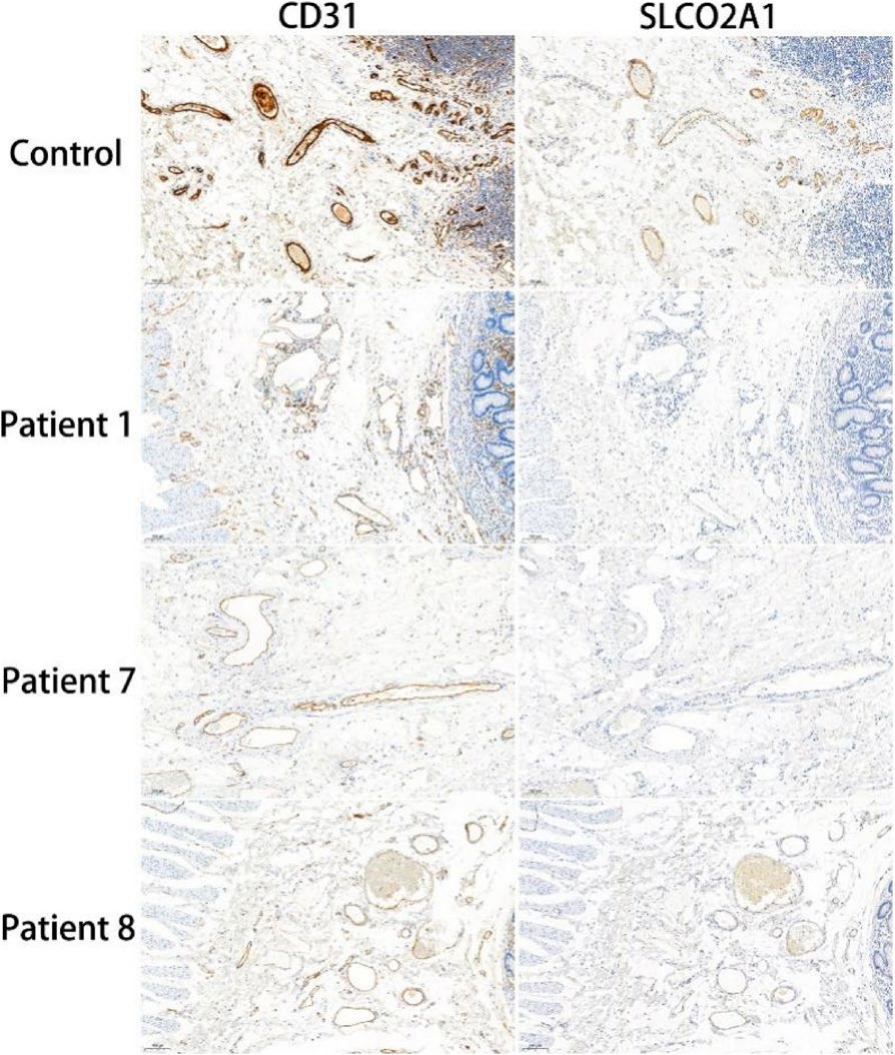
Immunohistochemical staining of CEAS surgical pathological sections. Representative immunohistochemical staining of anti-CD31 and anti-SLCO2A1 of intraoperative pathological sections from patient 1, patient 7, patient 8 and negative control without CEAS respectively (scale bar, 100 μm)

Surgical pathological samples of 6 CEAS patients who underwent surgeries in Peking Union Medical College Hospital were collected. Various extents of mucosal destruction characterized by multiple superficial ulcers in mucosa and submucosa, vasodilation and hyperemia in mucosal layer especially surrounding ulcers, and submucosal fibrotic hyperplasia were observed (Fig. 3). Statistically, multiple superficial ulcers involving mucosa and submucosa were present in all 6 patients. Blood vessel dilation and congestion were shown in 4 patients (66.7%), and submucosal fibrotic proliferation was observed in 5 patients (83.3%) (Table 1). Immunohistochemical staining revealed that in negative control SLCO2A1 was primarily expressed on vascular endothelium that was CD31-positive throughout the GI tract (Fig. 4). In CEAS patients, however, SLCO2A1 expression was significantly reduced (for example, patient 8) or absent (for example, patient 1 and 7) in vascular endothelial cells in mucosa and submucosa areas (Fig. 4).

### Genetic analysis

Sanger sequencing revealed genetic characteristics of the 12 CEAS patients, emphasizing on features of *SLCO2A1* mutations in particular (Table 2). All of the 12 patients showed *SLCO2A1* variants with base changes at various sites. Meanwhile, the types of mutations were diverse, such as missense, frame shift, etc. Of these 12 patients, 7 had homozygous mutations, 4 had compound heterozygous mutations (referring to two different mutant alleles on the same locus of two homologous chromosomes in one individual), and 1 had a heterozygous mutation. Patient 9-12 whose parents were consanguineous (Supplementary Table 1) all showed homozygous *SLCO2A1* variants. Altogether, fifteen different *SLCO2A1* variants were detected in the 12 patients. Polyphen2, ClinVar, MutationTaster were used respectively to predict the pathogenesis of these variants. All except the mutation of patient 10 were predicted as “probably damaging”, “likely pathogenic” or “disease causing” by at least one tool. Nevertheless, the mutation of patient 10, c1106G>A has previously been reported in PHO patients, suggesting the pathogenicity of this mutation [13]. In comparison with existing case reports of CEAS, 11 of the 15 variants detected from these 12 patients, whether homozygous or heterozygous, had not been reported previously in CEAS patients. Interestingly, 2 of the 11 newly discovered variants, c929G>A (homozygous mutation of patient 3) and c621C>A (1 mutant allele of patient 7) had not been previously reported, even in HGMD. This suggests the high diversity of *SLCO2A1* variants in CEAS patients and that the susceptibility of the SLCO2A1 protein to impaired function due to these mutations. However, when we looked at the clinical presentation of each patient and his or her genetic variants, there seemed no specific correlation between base change sites, mutation types and clinical phenotypes.

### Treatment

Symptomatic treatment was commonly used for these patients but showed limited effects. For instance, 7 (58.3%) patients took prednisone without effect (Table 1 and Supplementary Table 1). Four (33.3%) patients were treated with etoricoxib, which showed unsatisfactory effect. Given the patients’ nutritional deficiency status and iron deficiency anemia, enteral nutrition and iron supplementation were implemented in 9 (75.0%) patients and 12 (100.0%) patients respectively as supportive treatment. Seven (58.3%) patients were treated with mesalazine which was ineffective in 6 patients but effective for colorectal lesions in one patient. Thalidomide was ineffective in 1 patient and partially effective in another patient. Two (16.7%) patients received endoscopic balloon dilation due to stenosis of small intestine and colon. One patient was currently being treated with Tripterygium glycosides (a Chinese herbal extract with known anti-inflammatory and immunomodulatory effects empirically used by clinicians here to try to try to relieve the enteropathy), but the effect was uncertain so far.

Due to the unsatisfactory effect of medications mentioned above, surgery remains the primary effective treatment for CEAS patients. Nine (75.0%) patients had at least one abdominal surgery in the past to relieve symptoms and improve quality of life (Table 1 and Supplementary Table 1). Six (50.0%) patients had 1 surgery, while 3 (25.0%) had 2 surgeries. Partial ileal resection was performed in 8 (66.7%) patients, and subtotal gastrectomy in 2 (16.7%) patients. The median interval from CEAS symptom onset to surgery was 10 (IQR, 6-14) years.

## Clinical and genetic characteristics of Chinese patients diagnosed with chronic enteropathy associated with *SLCO2A1* geneDiscussion

CEAS, as a rare *SLCO2A1* gene-related gastrointestinal disease, is still not fully and deeply understood by clinicians as well as researchers, from its clinical features in various populations to its pathogenesis. Therefore, in this study, we reported the clinical, pathological and genetic characteristics of 12 Chinese patients diagnosed with CEAS. Some newly discovered clinical features and gene variants in Chinese CEAS cases were highlighted. Hopefully these new research findings will help improve our understanding of the common and diverse manifestations of this disease, especially among Chinese patients, and will facilitate future research on the pathogenic mechanisms.

Consistent with previous studies revealing severely delayed diagnosis, [4, 5] the CEAS patients in this study showed a long interval from symptom onset to confirmed diagnosis. Multiple reasons might contribute to this diagnostic delay, for example late definition of CEAS and complicated differential diagnosis of this disease with Crohn’s disease for instance [14, 15]. Aligning with previous reports on family history of CEAS, [4, 5] in our study 3 of 12 patients had a family history of PHO, another disease caused by *SLCO2A1* loss-of-function mutations, though none reported family history of CEAS. Besides, 33.3% of patients had parental consanguinity. The rate of parental consanguinity is similar to that of a previous Japanese case report [5]. Parental consanguinity is known to increase the probability of offspring to inherit homozygous variants and showing a recessive pathogenic phenotype. Genetic sequencing confirmed that these 4 patients with parental consanguinity indeed inherited homozygous SLCO2A1 variants, suggesting that their parents each carried at least one chromatid with the same variant.

On the other hand, in our study 7 of 12 CEAS patients were males who were all suffering from PHO-related symptoms in the meantime. The male-to-female ratio in our study was 1.4, which is much higher than previous studies with male-to-female ratios less than 1 [4, 5, 16]. However, it would be improper to generalize this sex ratio to be universally applicable. Given that Peking Union Medical College Hospital is a national center of bone metabolic disease, it is reasonable that male CEAS patients with PHO symptoms would prefer to come to this comprehensive medical center, so males with PHO takes up a higher proportion in this study. One inevitable limitation of this study is the small sample size given the rarity of this disease. Thus, further validation is needed when generalizing some data, results or conclusions. Nevertheless, we conducted long follow-ups of these patients and collected clinical data as thoroughly as possible to comprehensively analyze the characteristics of CEAS while ensuring data quality.

In terms of clinical symptoms, abdominal pain, hypoalbuminemia and anemia were common among CEAS patients, which is consistent with previous studies [4]. Gastrointestinal bleeding such as melena and hematochezia was more common in this study than previous reports [4]. Gastrointestinal lesions may cause their abdominal pain. Absorption disorders of nutrition ingredients such as protein, vitamin and iron, and persistent gastrointestinal bleeding may lead to hypoalbuminemia and anemia in CEAS patients. Long-term malabsorption of nutrients, anemia and hypoalbuminemia may further contribute to their low BMI. Another fact that catches our attention is that 4 of the 5 female patients experienced early-onset amenorrhea. Since there is a strong gender bias in PHO and CEAS with common causative *SLCO2A1* mutations, we propose that SLCO2A1 might exert some sex-specific effects in different tissues through the act of PGs, thus altering the female hormones or affecting menstruation. PGs are known to play important roles in female reproductive function, with PG transporters (PGT) expressed in uterine mediating the action of PG during menstrual cycle and pregnancy [17, 18]. Indeed, studies with human tissues revealed that expression of PGT in endometrium is modulated during menstrual cycle, and is increased in endometriosis patients, suggesting potentially essential physiological and pathological roles of SLCO2A1 in endometrium in females [19, 20]. Besides, the poor nutrition status and low BMI of the CEAS patients may further contribute to the development of amenorrhea. Despite the clinical observation and speculative mechanism mentioned above, the exact causal relationship between *SLCO2A1* mutations and the amenorrhea phenotype remains to be tested and studied with carefully designed experiments.

Regarding the lesion site, consistent with previous studies demonstrating that CEAS mostly affects ileum, [4–6] our endoscopic examinations revealed multiple ileal strictures with annular ulcer in most patients. However, gastric and colonic involvement was much more common in our patients compared with previous studies, suggesting that CEAS affects extensive parts along digestive tract. For instance, pyloric obstruction and sigmoid colon stricture with ulcer were rare in previous reports, but were present in 16.7% of the patients in our study respectively. A number of reasons may lead to the different proportions of different lesion sites. For example, different populations live in different environments, have different genetic backgrounds, lifestyles and dietary habits, etc. Also, the sample sizes of aforementioned studies and this study are limited to generalize this conclusion. In addition, we consider that the presence of colorectal lesions indicating UC ((diffuse mucosal erosions in colon and rectum) in one patient (patient No.3, Table 1 and Supplementary Table 1) to be a concurrent comorbidity with CEAS, rather than being caused by the gene mutation.

The pathological analysis showed multiple superficial ulcers, which is also the first identified pathological trait of CEAS or CNSU. Noteworthily, our pathological analysis revealed blood vessel dilation in mucosa and submucosa, as well as submucosal fibrotic proliferation in CEAS patients. Although the pathologic features of vasodilation and fibrotic proliferation were present simultaneously, the causal relationship between vasodilation and fibrotic proliferation and how SLCO2A1 loss-of-function causes these pathologic changes in digestive tract remain unclear. IHC results further revealed major SLCO2A1 loss in vascular endothelial cells in mucosa and submucosa areas of CEAS samples, which corresponds to previous studies showing high expression of SLCO2A1 in endothelial cells under physiological conditions [15, 21]. IHC staining of SLCO2A1 can be a good diagnostic tool besides DNA sequencing to detect impaired SLCO2A1 expression. Here we hypothesize that *SLCO2A1* loss-of-function mutations might cause vascular function alterations or ischemia, leading to vasodilation and fibrotic proliferation in intestinal lesions, which hopefully may provide clues to the future pathogenic mechanism of CEAS. Further research is needed to decipher the mechanism of SLCO2A1 regulation of vascular endothelial cells and fibroblasts and the crosstalk between various cell types in the pathogenesis of intestinal lesions.

In terms of genetic analysis, all 12 patients showed 15 different variants of *SLCO2A1* gene. Some variants have been widely reported in other populations. For example, patient 1 had homozygous variants c.1807C>T, which has been commonly reported in Japanese and Korean cases [4, 6] 11 variants were newly discovered for CEAS in this study. Two variants with c929G>A and c621C>A base changes, have not been reported pathogenic to any disease in HGMD database. Though all variants were detected from CEAS patients and most variants were predicted to be likely pathogenic by at least one tool, carefully designed experiments are still needed to verify that these novel genetic variants indeed cause protein loss-of-function and thus various degrees of pathogenicity. In this study, it is still inspiring that a number of new *SLCO2A1* variants were identified in Chinese CEAS patients, suggesting that the function or structure of SLCO2A1 protein can be easily disturbed or impaired by mutations at various sites and that there is a high diversity of genetic variants in CEAS patients.

For CEAS, the current medications are mainly for supportive and symptomatic treatment, and few drugs showed satisfactory effect. Although surgery remains the primary effective treatment available so far, it should be noted that resection of the diseased segments did not fundamentally prevent the onset and recurrence of CEAS symptoms along the whole GI tract. Researchers still need to further study the pathogenesis of CEAS in order to find proper targeted treatment and prevention methods for patients with *SLCO2A1* loss-of-function mutations.

## Conclusions

In conclusion, this study presents characteristics of 12 Chinese CEAS patients, highlighting symptoms and pathological features that are previously unreported or different from previous cases. Of note, 11 new *SLCO2A1* variants for CEAS are identified. Our study is a very strong addition to the current understanding of the disease.

### Supplementary Information

**Additional file 1: Supplementary Table 1.** Detailed Clinical Manifestations and Examinations of 12 CEAS patients.

## Data Availability

Data from public database are available on Human gene mutation database (HGMD, https://www.hgmd.cf.ac.uk/ac/index.php). Research data are incorporated into the article and its supplementary material, and the data underlying this article will be shared on reasonable request to the corresponding author.

## References

[1] Matsumoto T, Iida M, Matsui T (2004). Non-specific multiple ulcers of the small intestine unrelated to non-steroidal anti-inflammatory drugs. J Clin Pathol.

[2] Umeno J, Hisamatsu T, Esaki M (2015). A Hereditary Enteropathy Caused by Mutations in the SLCO2A1 Gene Encoding a Prostaglandin Transporter. PLoS Genet.

[3] Zhang Z, Xia W, He J (2012). Exome sequencing identifies SLCO2A1 mutations as a cause of primary hypertrophic osteoarthropathy. Am J Hum Genet.

[4] Hong HS, Baek J, Park JC (2022). Clinical and Genetic Characteristics of Korean Patients Diagnosed with Chronic Enteropathy Associated with SLCO2A1 Gene: A KASID Multicenter Study. Gut Liver.

[5] Umeno J, Esaki M, Hirano A (2018). Clinical features of chronic enteropathy associated with SLCO2A1 gene: a new entity clinically distinct from Crohn's disease. J Gastroenterol.

[6] Hosoe N, Ohmiya N, Hirai F (2017). Chronic Enteropathy Associated With SLCO2A1 Gene [CEAS]-Characterisation of an Enteric Disorder to be Considered in the Differential Diagnosis of Crohn's Disease. J Crohns Colitis.

[7] Jimbo K, Okuno T, Ohgaki R (2020). A novel mutation in the SLCO2A1 gene, encoding a prostaglandin transporter, induces chronic enteropathy. PLoS One.

[8] Jeong B, Park SH, Ye BD (2023). A Novel Chronic Enteropathy Associated with SLCO2A1 Gene Mutation: Enterography Findings in a Multicenter Korean Registry. Korean J Radiol.

[9] Seki S, Tanaka G, Kimura T (2022). Functional analysis of mutant SLCO2A1 transporters found in patients with chronic enteropathy associated with SLCO2A1. J Gastroenterol Hepatol.

[10] Wang Q, Li YH, Lin GL (2019). Primary hypertrophic osteoarthropathy related gastrointestinal complication has distinctive clinical and pathological characteristics: two cases report and review of the literature. Orphanet J Rare Dis.

[11] Wang Q, Xu H, Li Y (2021). Clinical and genetic characteristics of patients with chronic enteropathy associated with SLCO2A1 gene. Zhonghua Nei Ke Za Zhi.

[12] Hamon A, Cazals-Hatem D, Stefanescu C (2022). Crohn-like disease affecting small bowel due to monogenic SLCO2A1 mutations: First cases of Chronic Enteropathy Associated with SLCO2A1 gene (CEAS) in France. J Crohns Colitis.

[13] Zhang Z, He JW, Fu WZ (2013). Mutations in the SLCO2A1 gene and primary hypertrophic osteoarthropathy: a clinical and biochemical characterization. J Clin Endocrinol Metab.

[14] Yanai S, Yamaguchi S, Nakamura S (2019). Distinction between Chronic Enteropathy Associated with the SLCO2A1 Gene and Crohn's Disease. Gut Liver.

[15] Yamaguchi S, Yanai S, Nakamura S (2018). Immunohistochemical differentiation between chronic enteropathy associated with SLCO2A1 gene and other inflammatory bowel diseases. Intest Res.

[16] Oba MS, Murakami Y, Nishiwaki Y (2021). Estimated Prevalence of Cronkhite-Canada Syndrome, Chronic Enteropathy Associated With SLCO2A1 Gene, and Intestinal Behcet's Disease in Japan in 2017: A Nationwide Survey. J Epidemiol.

[17] Banu SK, Arosh JA, Chapdelaine P (2003). Molecular cloning and spatio-temporal expression of the prostaglandin transporter: a basis for the action of prostaglandins in the bovine reproductive system. Proc Natl Acad Sci U S A.

[18] Niringiyumukiza JD, Cai H, Xiang W (2018). Prostaglandin E2 involvement in mammalian female fertility: ovulation, fertilization, embryo development and early implantation. Reprod Biol Endocrinol.

[19] Kang J, Chapdelaine P, Parent J (2005). Expression of human prostaglandin transporter in the human endometrium across the menstrual cycle. J Clin Endocrinol Metab.

[20] Rakhila H, Bourcier N, Akoum A (2015). Abnormal Expression of Prostaglandins E2 and F2alpha Receptors and Transporters in Patients with Endometriosis. Biomed Res Int.

[21] Topper JN, Cai J, Stavrakis G (1998). Human prostaglandin transporter gene (hPGT) is regulated by fluid mechanical stimuli in cultured endothelial cells and expressed in vascular endothelium in vivo. Circulation.

