# Supplementary material for: Clinical and genetic characteristics of Chinese patients diagnosed with chronic enteropathy associated with SLCO2A1 gene
Source: Orphanet J Rare Dis. 2024 May 16;19:201. doi: 10.1186/s13023-024-03177-y (PMC11100163; doi:10.1186/s13023-024-03177-y)
Supplement: Supplementary file 1 — Additional file 1: Supplementary Table 1. Detailed Clinical Manifestations and Examinations of 12 CEAS patients. [file 13023_2024_3177_MOESM1_ESM.docx]

**Supplementary Table 1** Detailed Clinical Manifestations and Examinations of 12 CEAS patients

| **Indexes** | **Patient 1** | **Patient 2** | **Patient 3** | **Patient 4** | **Patient 5** | **Patient 6** | **Patient 7** | **Patient 8** | **Patient 9** | **Patient 10** | **Patient 11** | **Patient 12** |
| --- | --- | --- | --- | --- | --- | --- | --- | --- | --- | --- | --- | --- |
| Gender | M | M | M | M | M | M | F | F | M | F | F | F |
| Age at CEAS diagnosis, yr | 32 | 39 | 27 | 26 | 31 | 47 | 28 | 42 | 29 | 47 | 32 | 34 |
| Age at CEAS symptom onset, yr | 20 | 29 | 25 | 18 | 5 | 17 | 7 | 31 | 24 | 11 | 8 | 24 |
| Interval from symptom onset to diagnosis, yr | 12 | 10 | 2 | 8 | 26 | 30 | 21 | 11 | 5 | 36 | 24 | 10 |
| PHO diagnosis | + | + | + | + | + | + | - | - | + | - | - | - |
| Past NSAIDs use | + | - | - | + | - | + | - | - | + | + | - | - |
| Family history of CEAS | - | - | - | - | - | - | - | - | - | - | - | - |
| Family history of PHO | - | - | - | - | - | - | - | - | + | + | - | + |
| Parental consanguinity | - | - | - | - | - | - | - | - | + | + | + | + |
| **CEAS Symptoms** |  |  |  |  |  |  |  |  |  |  |  |  |
| Ileus | + | + | - | - | - | - | + | + | + | + | + | + |
| Diarrhea | + | + | + | - | - | - | - | + | - | - | - | + |
| Melena | - | - | - | + | + | - | - | - | - | + | + | - |
| Hematochezia | + | - | + | - | - | - | + | - | - | + | - | - |
| Fever | - | - | + | - | - | - | - | - | - | + | - | - |
| Pyloric obstruction | - | - | - | - | + | + | - | - | - | - | - | - |
| BMI, kg/m^2^ | 17.9 | 16.8 | 23.4 | 20.5 | 19.0 | 16.8 | 17.9 | 17.8 | 20.1 | 15.4 | 16.8 | 16.2 |
| **GI tract distribution** |  |  |  |  |  |  |  |  |  |  |  |  |
| Esophagus | + | - | - | - | - | + | - | - | - | - | - | - |
| Stomach | + | - | + | + | + | + | - | - | + | - | - | - |
| Small Intestine | + | + | - | + | + | + | + | + | + | + | + | + |
| Duodenum | + | - | - | + | + | + | - | - | - | - | - | - |
| Jejunum | + | - | - | - | - | - | + | - | - | - | - | - |
| Ileum besides terminal ileum | + | + | - | + | + | + | + | + | + | + | + | + |
| Terminal ileum | - | + | - | - | - | + | + | + | - | + | + | - |
| Colon | + | - | + | - | - | - | - | - | - | + | - | - |
| Rectum | **-** | **-** | **+** | **-** | **-** | **-** | **-** | **-** | **-** | **-** | **-** | **-** |
| **Endoscopic manifestations** |  |  |  |  |  |  |  |  |  |  |  |  |
| Reflux esophagitis | **+** | **-** | **-** | **-** | **-** | **+** | **-** | **-** | **-** | **-** | **-** | **-** |
| Gastric polyps | **+** | **+** | **+** | **+** | **-** | **-** | **-** | **-** | **-** | **+** | **-** | **-** |
| Hypertrophic gastritis | **-** | **-** | **+** | **-** | **-** | **-** | **-** | **-** | **+** | **-** | **-** | **-** |
| Gastric anastomotic ulcer | **-** | **-** | **-** | **-** | **+** | + | **-** | **-** | **-** | **-** | **-** | **-** |
| Ileal multiple strictures with annular ulcer | **+** | **+** | **-** | **+** | **+** | **+** | **+** | **+** | **+** | **+** | **+** | **+** |
| Sigmoid colon isolated stricture with annular ulcer | **+** | **-** | **-** | **-** | **-** | **-** | **-** | **-** | **-** | **+** | **-** | **-** |
| Diffuse mucosal erosions in colon and rectum | **-** | **-** | **+** | **-** | **-** | **-** | **-** | **-** | **-** | **-** | **-** | **-** |
| **Surgical pathology manifestation** |  |  |  |  |  |  |  |  |  |  |  |  |
| Multiple superficial ulcers | **+** | **+** | **/** | **/** | **/** | **/** | **+** | **+** | **/** | **+** | **+** | **/** |
| Blood vessel dilation and congestion | **+** | **+** | **/** | **/** | **/** | **/** | **-** | **+** | **/** | **/** | **+** | **/** |
| Submucosal fibrotic proliferation | **+** | **+** | **/** | **/** | **/** | **/** | **+** | **+** | **/** | **/** | **+** | **/** |
| **History of abdominal surgery** |  |  |  |  |  |  |  |  |  |  |  |  |
| Times of abdominal surgery | 2 | 2 | 0 | 0 | 2 | 1 | 1 | 1 | 1 | 1 | 1 | 0 |
| Sites of abdominal surgery |  |  | / | / |  |  |  |  |  |  |  | / |
| Stomach | / | / |  |  | + | + | / | / | / | / | / |  |
| Ileum | + | + |  |  | + | / | / | + | + | + | + |  |
| Ileocecum | / | / |  |  | / | / | + | / | / | / | / |  |
| Ileum-colon | + | / |  |  | / | / | / | / | / | / | / |  |
| Duration from CEAS symptoms onset to surgery, yr | 10 | 6 | / | / | 15 | 10 | 21 | 1 | 1 | 8 | 14 | / |
| **Laboratory Tests** |  |  |  |  |  |  |  |  |  |  |  |  |
| Lowest hemoglobin, g/L | 44 | 82 | 96 | 73 | 59 | 81 | 37 | 51 | 90 | 60 | 43 | 73 |
| Lowest albumin, g/L | 14 | 25 | 34 | 42 | 28 | 22.8 | 27.2 | 10.5 | 43 | 30 | 21 | 32 |
| CRP, mg/L | 50.6 | / | 23.4 | 17.0 | 12.2 | 3.5 | 6.4 | 11.9 | 51.7 | 7.0 | 46.3 | 1.73 |
| Platelet, 10^9^/L | 418 | 204 | 566 | 130 | 270 | 194 | 429 | 513 | 158 | 135 | 253 | 264 |
| ASCA | + | - | - | - | + | - | - | - | - | - | - | - |
| ANCA | - | - | + | - | - | - | - | - | - | - | - | - |
| **Other diseases** | Congenital lymphatic dysplasia | - | - | - | / | Chole-lithiasis | Ameno-rrhea | Amenorrhea; Hypo-thyroidism | - | Ameno-rrhea | Ameno-rrhea | Lymphoid tuberculosis |
| **Treatment history** |  |  |  |  |  |  |  |  |  |  |  |  |
| Prednisone | Ineffective | Ineffective | / | / | / | / | / | Ineffective | Ineffective | Ineffective | Ineffective | Ineffective |
| Etoricoxib | Unsatisfactory | / | / | Unsatisfactory | / | Unsatisfactory | / | / | Unsatisfactory | / | / | / |
| Enteral nutrition | + | + | / | / | + | + | + | + | / | + | + | + |
| Iron supplementation | + | + | + | + | + | + | + | + | + | + | + | + |
| Mesalazine | / | / | Effective for colorectal lesions | / | Ineffective | / | Ineffective | Ineffective | / | Ineffective | Ineffective | Ineffective |
| Endoscopic balloon dilation | + | / | / | / | / | + | / | / | / | / | / | / |
| Thalidomide | Ineffective | / | / | / | / | / | / | / | / | / | Partially effective |  |
| Others |  |  |  |  |  |  |  |  |  |  |  | Tripterygium glycosides in use. The effect was uncertain. |

CEAS, chronic enteropathy associated with SLCO2A1 gene; yr, year; M, male; F, female; PHO, primary hypertrophic osteoarthropathy; NSAIDs, nonsteroidal anti-inflammatory drugs; BMI, body mass index; GI, gastrointestinal; CRP, C-reactive protein; ASCA, anti-*Saccharomyces cerevisiae* antibodies; ANCA, anti-neutrophil cytoplasmic antibodies
